# Diverse Functions of Polyamines in Virus Infection

**DOI:** 10.3390/biom10040628

**Published:** 2020-04-18

**Authors:** Mason R. Firpo, Bryan C. Mounce

**Affiliations:** Department of Microbiology and Immunology, Stritch School of Medicine, Loyola University Chicago, Maywood, IL 60153, USA; mfirpo@luc.edu

**Keywords:** polyamines, viruses, host–virus interactions, metabolism

## Abstract

As obligate intracellular parasites, viruses rely on host cells for the building blocks of progeny viruses. Metabolites such as amino acids, nucleotides, and lipids are central to viral proteins, genomes, and envelopes, and the availability of these molecules can restrict or promote infection. Polyamines, comprised of putrescine, spermidine, and spermine in mammalian cells, are also critical for virus infection. Polyamines are small, positively charged molecules that function in transcription, translation, and cell cycling. Initial work on the function of polyamines in bacteriophage infection illuminated these molecules as critical to virus infection. In the decades since early virus-polyamine descriptions, work on diverse viruses continues to highlight a role for polyamines in viral processes, including genome packaging and viral enzymatic activity. On the host side, polyamines function in the response to virus infection. Thus, viruses and hosts compete for polyamines, which are a critical resource for both. Pharmacologically targeting polyamines, tipping the balance to favor the host and restrict virus replication, holds significant promise as a broad-spectrum antiviral strategy.

## 1. Polyamine Synthesis and Regulation

**Figure 1:**
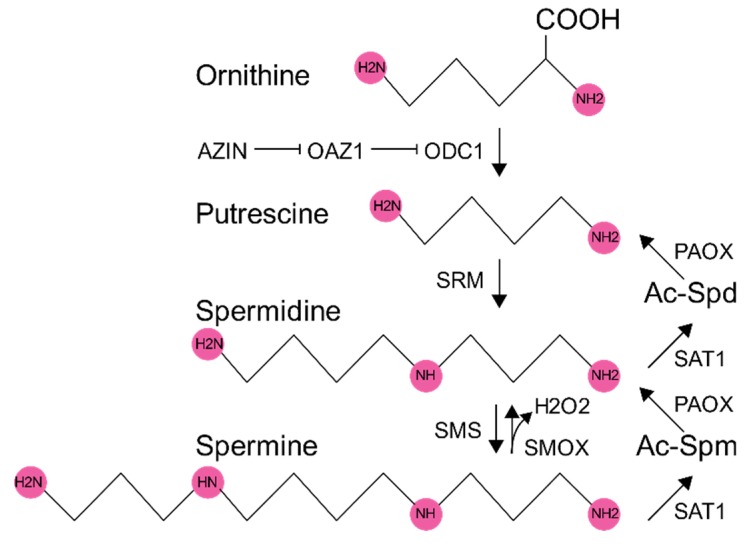
Schematic of the polyamine pathway in mammalian cells. An ornithine precursor is deacetylated by ornithine decarboxylase (ODC1) to form putrescine. ODC1 is inhibited through the action of ODC1 antizyme (OAZ1), which is inhibited by antizyme inhibitor (AZIN), in turn. Putrescine is converted to spermidine by spermidine synthase (SRM), and spermidine is further converted to spermine by spermine synthase (SMS). The conversion of spermine to spermidine releases hydrogen peroxide (H_2_O_2_) by the action of spermine oxidase (SMOX). Spermine and spermidine are acetylated by spermidine–spermine acetyltransferase (SAT1) to form acetylated moieties (Ac-Spm and Ac-Spd). Acetylated spermidine or spermine can be converted to putrescine or spermidine, respectively, through the action of polyamine oxidase (PAOX).

Polyamines are small, abundant, flexible, biomolecules that consist of carbon chains containing amino groups that give them a positive charge at cellular pH. Eukaryotes have three polyamines that are synthesized in a stepwise process from arginine. Arginine is converted to ornithine, which is decarboxylated via ornithine decarboxylase 1 (ODC1) to putrescine. Putrescine is converted to spermidine via spermidine synthase (SRM) followed by conversion to spermine via spermine synthase (SMS). The overview of this pathway can be seen in Figure 1. Spermine and spermidine can be catabolized back to putrescine by the addition of an acetyl group by spermidine/spermine acetyltransferase (SAT1) followed by polyamine oxidase (PAOX). Spermine can also be catabolized back to spermidine via spermine oxidase (SMOX). Polyamine synthesis and degradation are tightly controlled metabolic processes in which polyamines regulate their own synthesis (reviewed in [1]). ODC-1 antizyme (OAZ1) regulates the turnover of ODC1, in which OAZ1 translation is regulated by a frameshifting mechanism that is polyamine dependent. OAZ1 itself is inhibited by antizyme (AZIN). SAMDC, an enzyme that is crucial for the conversion of putrescine to spermidine and spermine is regulated by an upstream ORF (uORF), at which ribosomes pause prior to the stop codon, stalling scanning ribosomes from initiating the translation of SAMDC [2]. Spermidine and spermine levels enhance this pause; thus, high levels of these molecules precludes their synthesis. Finally, SAT1 activity, transcription, and translation is regulated by polyamines, with high polyamine content leading to enhanced SAT1 levels and activity (reviewed in [3]). ODC and SAMDC can also be inhibited by nitric oxide (NO) production through the nitroslyation of a cysteine in the active site [4] (reviewed in [5]). Overall, polyamine synthesis is a highly regulated process, and the cell expends considerable energy in maintaining homeostatic polyamine levels.

**Figure 2:**
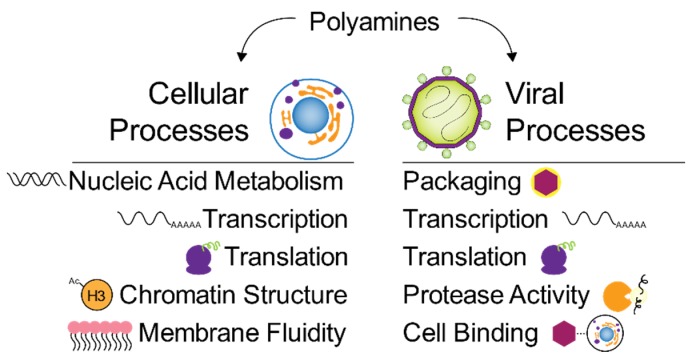
Polyamines in cellular and viral processes. Cells rely on polyamines for transcription, translation, nucleic acid metabolism and structure, chromatin and DNA packaging, and membrane fluidity. Viruses similarly utilize polyamines for transcription, translation, and nucleic acid packaging, but they also use polyamines in binding to cells and for enhancing virus enzyme activity.

Putrescine, spermidine, and spermine all play key roles in a wide variety of cellular processes (Figure 2). Due to their charge, they bind nucleic acids as well as affect chromatin structure [6]. In brain tumor cells depleted of polyamines, chromatin condensation was negatively affected [7]. Polyamines also promote the Z-DNA conformation and stabilize the DNA quadruplex conformation of c-myc, resulting in the overexpression of c-myc [8,9]. This acts as a positive feedback loop due to the ODC, SRM, and SMS genes being transcriptional targets of c-myc [10,11]. Spermidine has been shown to be essential for cell proliferation in eukaryotes, specifically for the hypusination of eIF5A, an initiation factor that regulates translation of a variety of cellular proteins. Hypusination is unique to eIF5A, and the current model of its function is in the translation of “hard-to-translate” regions of polypeptides, such as polyproline tracts. Recent work has shown that hypusination promotes autophagy in B cells [12] and the expression of oxidative phosphorylation proteins in macrophages [13]. Additionally, polyamines regulate membrane fluidity: positively charged polyamines interact with negatively charged phospholipid headgroups to reduce lipid movement. Polyamines also regulate ion channel function, protein–RNA interactions, and cell junctions, among others. All of these critical roles within the cell are also crucial for virus infection, suggesting that these molecules that are important for cellular functions also play roles in virus infection.

## 2. Polyamines in Bacteriophages

Some of the first studies on bacteriophages, the viruses of bacteria, involved polyamines and their roles in viral infection (Figure 2). In fact, bacteriophages were critical systems that first established a role for polyamines in virus infection. A subset of both DNA and RNA bacteriophages package polyamines within the virions. In bacteriophage R17, for instance, spermidine and putrescine were identified within virions, specifically associating with the viral RNA. Quantifying viral particles and polyamines, the authors estimated approximately 1000 polyamines per viral particle, enough to neutralize approximately 50% of the genome’s negative charge. The authors hypothesized that polyamines specifically functioned to neutralize such as putrescine and spermidine could be replaced with either spermine or Mg^2+^. Work on bacteriophage T4 demonstrated that *E. coli* growth conditions affect phage polyamine content. Phages harvested from agitated medium incorporated low levels of polyamines; however, phage isolated from anaerobic conditions incorporated cadaverine, which is a polyamine formed via lysine decarboxylation [14]. Thus, at least in the case of bacteriophage T4, the metabolic state of the infected cell affects polyamine incorporation in the virion. These phage-associated polyamines were largely considered to bind nucleic acids and enhance compaction, contributing significantly to phage stabilization [15].

Beyond packaging and neutralizing negatively charged genomes, work in bacteriophages also highlighted a role for polyamines in viral DNA replication. When bacteriophage T4 infects polyamine-depleted *E. coli* K-12, phage DNA synthesis and virion maturation were significantly reduced. Work with bacteriophage f2 using polyamine auxotrophic *E. coli* also demonstrated a role for polyamines in phage translation [16]. Bacteriophages also manipulate bacterial polyamine levels, presumably to enhance phage functions. For instance, bacteriophage R17 promotes spermidine accumulation in the infected *E. coli* cell [17,18]. However, this phenotype is not shared with all bacteriophages. For instance, bacteriophage T4 infection results in no change in cellular polyamine synthesis or accumulation [19,20]. This work in bacteriophages highlighted that polyamines were crucial regulators of viral processes in prokaryotes, and later work soon demonstrated a role for polyamines in eukaryotic viruses as well.

## 3. Polyamines in Plant Viruses and the Response to Infection

Investigation into the potential roles of polyamines in plant viruses shortly followed the early work on bacteriophages. Akin to select bacteriophages, plant viruses incorporate polyamines. For instance, purified turnip yellow mosaic virus (TYMV) contains spermidine and spermine at levels approximately twice the cellular concentration of these molecules [21,22,23,24]. When cellular polyamine pools were shifted to reduce spermidine levels, higher levels of spermine were incorporated into purified virions, suggesting flexibility in polyamine packaging for TYMV [21]. In contrast, belladonna mosaic virus (BDMV) incorporates putrescine and spermidine, potentially for maintaining virion stability at alkaline pH [25,26]. However, these polyamines could be replaced with potassium ions, although this resulted in reduced virion stability [25]. Several plant viruses, including turnip crinkle and tobacco mosaic virus incorporate the polyamine bis(3-aminopropyl)amine, which appears to be specifically present in infected but not healthy plants [22]. However, whether the incorporation of this modified polyamine is to the virus’ benefit is unclear. Further, whether packaged polyamines enhance virion stability or have other virion functions for these diverse viruses is unknown.

During viral infection, plant cells exhibit increased polyamine levels, as well as increased levels of polyamine-conjugated molecules. For example, in tobacco mosaic virus infection, polyamine levels rise [27]. However, these polyamines are rapidly degraded by polyamine oxidases, which are also upregulated in the tobacco plant upon virus infection. As a by-product of polyamine oxidation, hydrogen peroxide is produced, which induces cellular death and precludes virus replication [28], as part of the hypersensitive response. Interestingly, these phenotypes are observed only in plants that induce the hypersensitive response and not in plants that are susceptible to TMV infection. Much remains to be understood of precisely how polyamines function in virus infections of plants, however.

Although unrelated to viruses such as TMV or BDMV, the algae virus *Paramecium busaria* chlorella virus-1 (PBCV-1) is a large DNA virus encoding 331 kb and 365 (known) genes. This virus evolved to encode entire metabolic pathways, including a polyamine biosynthetic pathway [29]. The ornithine decarboxylase [30] and polyamine acetyltransferase [31] have been cloned, and their biochemical activity verified, thus identifying these as the first virally-encoded polyamine genes. Additionally, PBCV-1 encodes a homospermidine synthase molecule, in addition to a spermidine synthase enzyme, that produces homospermidine from two putrescine molecules [32]. However, the precise roles of these enzymes and the polyamines they produce are unknown, either during infection or in the context of the PBCV-1 virion. Further, whether other related viruses encode similar pathways or rely on host pathways for virus multiplication is unexplored. Thus, the implications for polyamines in algae–virus ecology are unclear.

## 4. Polyamines in Mammalian Viruses and the Response to Infection

From their key roles in mammalian cells, polyamines also play important roles in viral infection. Their importance and roles for viruses are diverse. To date, roles for polyamines in mammalian viruses include viral entry, transcription, replication, and virion packaging. While the earliest reports of polyamines in bacteriophages was in the late 1950s [33], it was not until 1971 that polyamines were found in a human virus. This virus was the herpes simplex virus (HSV-1), and it was found to contain spermine in the nucleocapsid and spermidine in the viral envelope [34]. Later, it was shown that human cytomegalovirus (HCMV) stimulated ODC1 activity and increased polyamine uptake, presumably to enhance cellular polyamine levels. The inhibition of polyamine metabolism after the eclipse phase did not affect viral replication, suggesting that polyamines may not play a role late in infection but may have roles early in infection [35,36,37].

The initial descriptions of spermidine and spermine in the virions of HSV-1 suggested that mammalian viruses may package polyamines, as observed for bacteriophages and plant viruses. HSV-1 was shown to produce comparable amounts of viral particles in cells depleted of polyamines with the specific ODC1 inhibitor DFMO and control cells, but those produced from polyamine-deficient cells had different abundance of DNA fragments [38] and DFMO-treated cells produced lower viral titers [39]. Similarly, the bunyaviruses, Rift Valley Fever virus, and Lacrosse virus produced non-infectious particles in polyamine-depleted cells [40]. Polyamines are present to varying degrees in capsids of diverse viruses. Vaccinia virus (VACV), a double-stranded DNA virus with a large (1 Mb) genome, packages polyamines putatively to neutralize DNA’s negative charge [34,41]. In contrast, poliovirus and Coxsackievirus package negligible amounts of polyamines [42]. The precise roles of these packaged polyamines are unclear.

**Figure 3:**
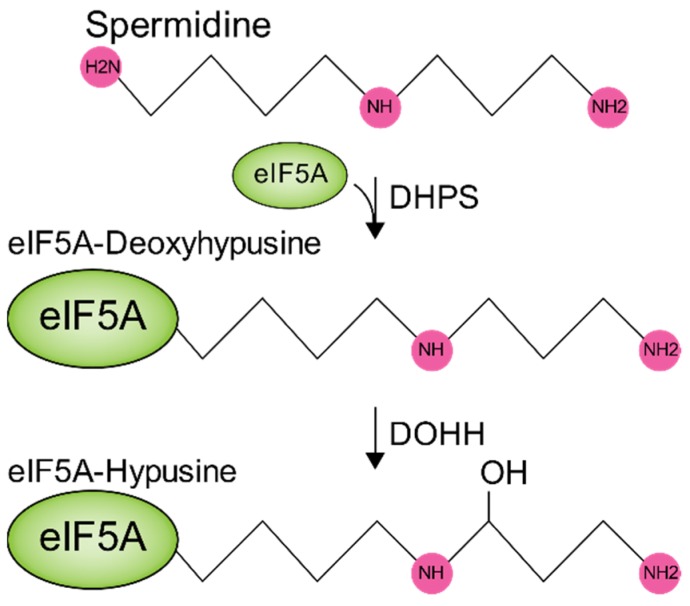
The mammalian eIF5A hypusination pathway. Spermidine is conjugated to eukaryotic initiation factor 5A (eIF5A) by the action of deoxyhypusine synthase (DHPS) to form eIF5A-deoxyhypusine, which is deacetylated by deoxyhypusine hydroxylase (DOHH) to form eIF5A-hypusine.

Early work in bacteriophages suggested a role for polyamines in polymerase activity and viral genome synthesis. Work with mammalian viruses also showed that polyamines contribute to these functions, specifically in the alphavirus chikungunya virus (CHIKV) and the flaviviruses Zika virus (ZIKV) [43], hepatitis C virus (HCV) [44], and the herpesvirus HSV-1 [45]. In vitro genome synthesis assays demonstrated that polyamines enhanced viral polymerase activity, similar to bacteriophage T7 polymerase [46]. In addition to roles in polymerase activity, CHIKV, ZIKV, and HCV translation rely on polyamines, as DFMO-treated (polyamine depleted via ODC1 inhibition) cells exhibited reduced viral translation [43,47]. Further work in Ebola virus (EBOV) and Marburgvirus (MARV) highlighted that polyamines function in transcription from viral genomes, but the translation of these transcripts relied on polyamines through eIF5A hypusination (Figure 3) [48,49]. The knockdown of eIF5A or inhibition of the enzymes involved in its hypusination resulted in significant decreases in the accumulation of viral proteins. Further, hypusination inhibition reduced EBOV and MARV infectious titers. In sum, these data suggest that polyamines function in the transcription and translation of mammalian viruses and also highlight opportunities to target viral replication through polyamines and hypusination.

In addition to roles in transcription and translation, polyamines affect additional viral enzymes and processes to enhance virus replication. The enterovirus, Coxsackievirus B3 (CVB3), requires polyamines for multiple stages in its infectious life cycle. CVB3 exhibited a reduced ability to bind polyamine-depleted cells, which is a phenotype shared with additional enteroviruses. Interestingly, CVB3 passaged in the presence of DFMO accumulated a mutation in the capsid protein VP3 at site Q234R, which increased the cellular attachment of CVB3 to polyamine-depleted cells [50]. Additionally, CVB3 proteases 2A and 3C exhibit sensitivity to polyamines, as polyamine depletion precludes their activity both in vitro and in the context of infection. CVB3 also showed resistance to polyamine depletion through mutations in its 2A and 3C proteases, which are responsible for cleaving host and viral proteins [51]. Curiously, mutations observed here and in a similar experiment with CHIKV changed a negatively charged amino acid to a positively charged residue (either lysine or arginine), as if to substitute for the loss of the positively charged polyamines.

Several additional viruses are sensitive to polyamine depletion. A broad screen of viruses [52] highlighted a role for polyamines in enterovirus, alphavirus, flavivirus, rhabdovirus, coronavirus, and bunyavirus infection. The precise roles of polyamines in these viruses remain to be completely understood, but this and others’ work highlights the potential for polyamine depletion as an antiviral strategy.

Upon infection, cellular signaling through the innate immune response stimulates the expression of hundreds of interferon-stimulated genes (ISGs) that restrict virus replication. Several of the ISGs are metabolic enzymes, including SAMHD1 and IDO1, which deplete nucleotides and tryptophan, respectively, to limit virus replication [53]. Given their importance in virus replication, polyamines are similarly depleted upon virus infection. Upon signaling through interferon α/β, SAT1 is induced and depletes cellular polyamines. In fact, SAT1 knockout cells replicate virus to higher titers than wild-type cells when treated with type I interferon. Prior work also highlighted that ODC1 activity decreases with interferon α, β, or γ treatment, although the effect on polyamine levels was not investigated [54]. In a separate study, the intraperitoneal delivery of interferon α and β inhibited ODC1 activity in mice. Thus, polyamine depletion is a strategy by which mammalian cells can reduce viral infection. Since transient polyamine depletion is well-tolerated by most cells, temporary polyamine depletion to limit infection may be a reasonable strategy to limit virus infection and maintain cellular viability.

## 5. Viral Manipulation of Polyamines

**Figure 4:**
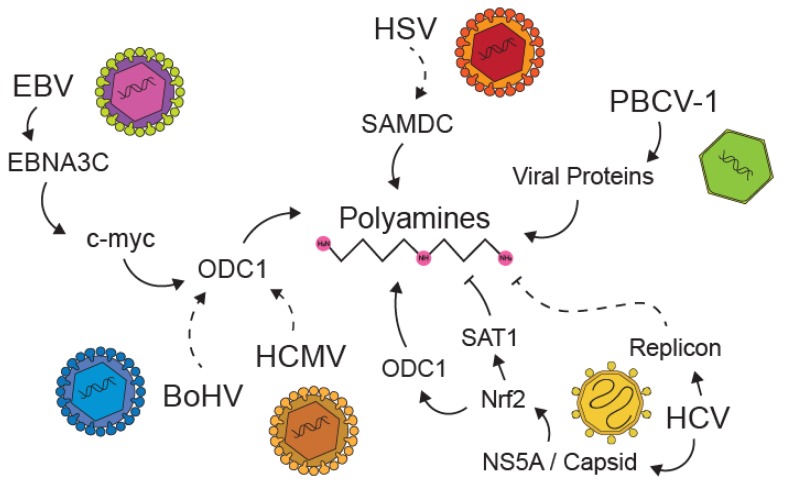
Viral manipulation of polyamines in infected cells. The herpesvirus Epstein–Barr virus (EBV), herpes simplex virus (HSV), bovine herpesvirus (BoHV), and human cytomegalovirus (HCMV) induce polyamine levels through ODC1 and SAMDC. Hepatitis C virus (HCV) proteins induce both SAT1 and ODC1, and cells harboring HCV replicons exhibit reduced levels of polyamines. *Paramecium busaria* chlorella virus (PBCV-1) encodes a polyamine biosynthetic pathway. Mechanisms that are not fully understood are represented by dashed lines.

Polyamines are important resources for viruses and cells, and viruses have evolved mechanisms to maintain, enhance, or manipulate polyamine metabolism to support virus infection (Figure 4). Perhaps the most extreme example is PBCV-1, described above, which encodes an entire polyamine biosynthesis pathway in its dsDNA genome [29]. This implies that polyamines are vital for these viruses to infect hosts; however, little is known about polyamines’ exact roles in infection. Epstein–Barr virus (EBV) has also been shown to manipulate polyamine levels in cells by decreasing the expression of SAT1, which is a polyamine catabolic enzyme that acetylates spermidine and spermine, causing them to be degraded or expelled out of the cell [55]. Additionally, EBV is also able to stabilize c-myc through its nuclear antigen 3C, resulting in overexpression of the polyamine synthesis proteins [56]. This overexpression of c-myc could be the cause of the downregulation of SAT1, but whether polyamines are directly involved in this process is still unknown. Herpes simplex virus (HSV-1) was also shown to upregulate the expression of SAMDC mRNA in infected cells [57] and human cytomegalovirus, another herpesvirus, stimulates ODC1 activity [35]. A distantly related virus, bovine herpes virus, encodes an ODC-like protein that shares around 55% amino acid homology with mammalian ODC and contained all the amino acids necessary for decarboxylase activity [58]. Whether additional viruses manipulate polyamine levels and the mechanisms involved therein is unknown. Interestingly, the viruses described above that manipulate polyamine levels are DNA viruses, and, in the case of herpesviruses, result in permanent infection of the host. Whether acute viruses, similar to many of the RNA viruses described above, affect polyamine levels remains to be explored. However, recent work has demonstrated that HCV induces polyamine metabolic genes, including ODC1, SAT1, and SMOX during infection, overall resulting in reduced polyamine levels in cells harboring an HCV replicon [59]. Whether this observation is true for other RNA viruses, particularly acute RNA viruses, is unknown; regardless, viruses have evolved strategies to interface with polyamines in infected cells.

## 6. Polyamines in Metabolic Pathways Key to Virus Infection

**Figure 5:**
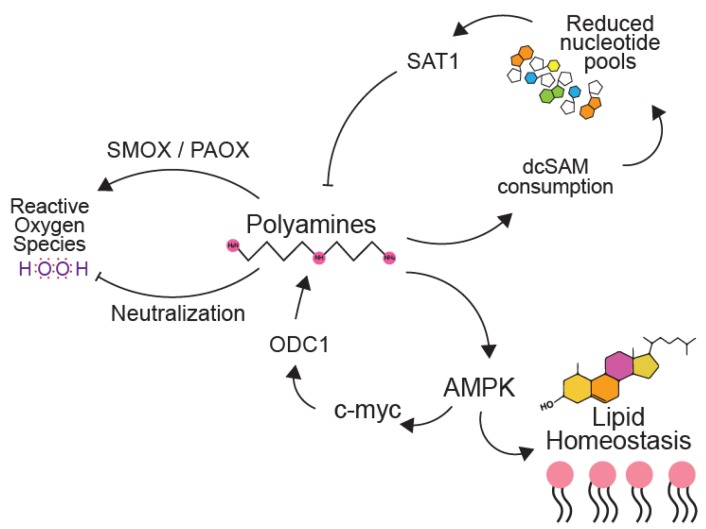
Polyamines affect diverse metabolic pathways in mammalian cells. Polyamine synthesis consumes decarboxylated S-adenosyl methionine (dcSAM), resulting in reduced nucleotide pools. These reduced nucleotide pools induce SAT1, which depletes polyamines, resulting in a feedback loop. The interconversion of polyamines via SMOX and PAOX accumulates reactive oxygen species, specifically hydrogen peroxide (H_2_O_2_). Polyamines also are described to neutralize reactive oxygen species due to their abundance and charge. Additionally, polyamines maintain AMPK in its unphosphorylated, non-activated state to maintain lipid homeostasis. AMPK activation induces polyamine synthesis through c-myc mediated ODC1 induction.

Cellular metabolic pathways are interconnected and complex. Polyamines play a wide role in key metabolic processes including nucleotide metabolism, the formation of reactive oxygen species (ROS), and lipid metabolism, among many other metabolic pathways described to interface in mammalian cells (Figure 5). Thus, polyamines likely affect virus replication through the modulation of these distinct cellular metabolic pathways. Polyamines play a role in nucleotide pools due to the requirement of decarboxylated S-adenosylmethionine (dcSAM) to act as the aminopropyl group donor. When cells were treated with DFMO, dcSAM was synthesized at normal levels, leading to higher levels of ATP/ADP as well as higher levels of the ribonucleotides UTP and CTP [60]. Polyamine depletion via DFMO also induces thymidine depletion in colon tumors [61]. Another link between polyamines and nucleotides was demonstrated in a study with the broad spectrum antiviral ribavirin, which resembles guanosine [62]. Treated cells exhibited higher levels of SAT1 and decreased polyamine levels; when guanosine was added exogenously to these treated cells, polyamine levels were restored, and viral titers were partially recovered [63]. This partial recovery of viral titers suggests that a portion of ribavirin’s antiviral activity is through polyamines and highlights the connectedness between nucleotide and polyamine synthesis.

The AMP-activated kinase (AMPK) is a broad regulator of cellular metabolism that, upon activation, decreases cellular lipid synthesis, enhances β-oxidation, increases glucose uptake, and activates autophagy. Cells treated with DFMO exhibit reduced AMPK activation [64]. When AMPK was knocked down in cardiomyoblasts in the presence of isoproterenol, a transcriptional activator of ODC1, ODC1 levels increased above those seen with treatment of isoproterenol alone, suggesting that AMPK is able to downregulate the transcription of ODC1 [65].

Polyamines and their catabolism play a part in the generation of ROS as well as sequestering ROS. The turnover of spermidine and spermine via PAOX results in the generation of hydrogen peroxide in cells. However, both spermidine and spermine have been shown to neutralize ROS and are important for the prevention of oxidative damage [66,67]. Interestingly, the SAT1 gene is targeted by p53, and SAT1 expression causes an increase in lipid peroxidation, stimulating cells to undergo ferroptosis [68]. Many viruses, including hepatitis C virus, herpes simplex virus, and influenza virus induce ROS formation during infection [69,70,71,72]. Polyamines may play a role in neutralizing these ROS, since their generation can cause a ferroptotic response and potentially draw unwanted attention from immune cells to the infected area. However, polyamines may not be beneficial for all viruses and may actually inhibit some stages of infection. The M2 protein of influenza, which plays a role in viral uncoating, can be inhibited by polyamines [73]. Influenza also causes an increase in NO, through interferon γ, which inhibits ODC1. Whether these processes aid influenza virus in subsequent cellular entry has yet to be explored.

## 7. Targeting Polyamines as an Antiviral Therapy

**Figure 6:**
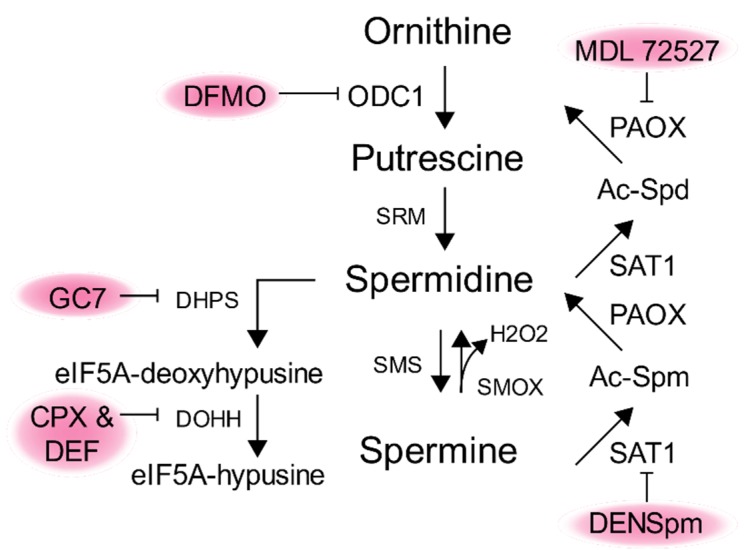
Polyamine-modulating molecules with antiviral activity. Several molecules target polyamine biosynthesis to alter polyamine levels and hypusinated eIF5A within the cell. Difluoromethylornithine (DFMO), MDL 72527, diethylnorspermidine (DENSpm), *N*^1^-guanyl-1,7-diamineheptane (GC7), ciclopirox (CPX), and deferiprone (DEF) target distinct metabolic enzymes and exhibit antiviral activity.

Given the importance of polyamines to diverse aspects of viral infection, polyamine synthesis inhibitors have gained attention as potential antivirals. Initially, targeting polyamines gained traction as a potential cancer therapeutic [74]. Given polyamines’ roles in promoting cell cycles, it was not surprising that several types of cancers enhanced polyamine synthesis. However, anti-cancer therapies targeting polyamines were largely unsuccessful initially. However, continued work on polyamines in the development and progression of cancer remains an active area of research. Importantly, several potential polyamine-targeting molecules (summarized in Figure 6) received extensive testing in animal models and clinical trials, providing a wealth of information on their toxicity and in vivo effects. Thus, these molecules may hold promise when repurposed as antivirals.

Perhaps the best known and characterized molecule is difluoromethylornithine (DFMO), which is an irreversible inhibitor of ODC1. DFMO treatment results in significant reductions in polyamine levels in a time- and dose-dependent manner in multiple cell types [75]. Clinically, DFMO is effective against trypanosomiasis, or African sleeping sickness, with mild side effects including reversible ototoxicity. The trypanosomal ODC1 is highly sensitive to DFMO, resulting in the reduction of polyamines in the parasite and clearance by infected individuals [76,77,78,79]. In fact, DFMO is a frontline drug in the treatment of trypanosomiasis and is on the list of the World Health Organization’s essential medicines [80]. DFMO can be taken orally, topically, or intravenously. Treated individuals experience mild side effects, including thrombocytopenia, although these effects are reversible upon the cessation of treatment [81]. DFMO inhibits infection by several viruses, both in vitro and in vivo [43,52]. Thus, DFMO administration may be a promising route to quell virus replication. However, virus infection is best reduced with DFMO pretreatment, and treatment post-exposure requires significant additional investigation. Nonetheless, prophylactic DFMO treatment may be reasonable in certain instances, such as to protect healthcare workers, uninfected contacts of infected patients, or immunocompromised individuals.

In addition to DFMO, other compounds have received attention as potential antivirals. Diethylnorspermidine (DENSpm), another potential anti-cancer therapeutic, enhances polyamine catabolism and rapidly depletes polyamines. Although not as extensively tested as DFMO, DENSpm has been explored in clinical trials [82,83]. DENSpm also exhibits broad antiviral activity and a shorter pretreatment time, making it potentially more functional than DFMO in certain instances. Additionally, molecules such as MDL 72757, targeting polyamine interconversion [84], exhibit antiviral activity in vitro [48]. Another highly promising category of molecules targeting polyamine metabolism are the hypusination inhibitors GC7, deferiprone, and ciclopirox. A recent review summarizes the promise of these molecules as antiviral agents [85]. Studies by Olsen and colleagues [48,49] demonstrated the efficacy of these inhibitors against Ebola virus infection, and prior work showed that HIV-1 is similarly sensitive to this group of inhibitors [86]. In sum, several polyamine-targeting molecules show activity against viruses, and further work into their mechanisms of action, toxicity, and in vivo activity is required for the possibility of targeting polyamines to treat or prevent viral infection.

## 8. Summary

Viruses evolve intimate relationships with host cells and their metabolites. While we are beginning to understand how viruses interface with metabolites within cells, much remains to be uncovered. A broad base of literature surrounding polyamines and viruses, from the 1950s to the present day, suggest that these small molecules have a big impact on virus infection. From initial descriptions of polyamines in bacteriophage virions to the elucidation of the mechanism by which eIF5A hypusination enhances Ebola virus replication, the breadth of our knowledge of polyamine-virus infection continues to expand. Additional descriptive and mechanistic research will uncover as-yet-unidentified mechanisms of polyamine function in infection of bacteria, plants, and mammals. Despite their simplicity, the complexity of polyamines in cells and viruses will continue to present fascinating basic and translational scientific questions.
